# Influence of Lactose on the Maillard Reaction and Dehydroalanine-Mediated Protein Cross-Linking in Casein and Whey

**DOI:** 10.3390/foods11070897

**Published:** 2022-03-22

**Authors:** Søren D. Nielsen, Lotte J. Knudsen, Line T. Bækgaard, Valentin Rauh, Lotte B. Larsen

**Affiliations:** 1 Department of Food Science, Aarhus University, Agro Food Park 48, 8200 Aarhus N, Denmark; ljk@food.au.dk (L.J.K.); line_baekgaard@hotmail.com (L.T.B.); lbl@food.au.dk (L.B.L.); 2 Arla Foods Innovation Centre, Agro Food Park 19, 8200 Aarhus N, Denmark; varau@arlafoods.com

**Keywords:** multiple reaction monitoring, advanced glycation end products, glycation, protein modification, milk, processing

## Abstract

A liquid chromatography–mass spectrometry method based on multiple reaction monitoring (MRM) was developed for the simultaneous quantification of markers representing two potentially competing pathways, the Maillard reaction and the dehydroalanine pathway. The two pathways involve the same residues in the proteins to some extent, namely, the essential amino acid lysine, as well as free-amino terminals available on proteins and polypeptides, competition between the two pathways in food systems may occur. The developed method comprises the following markers of the Maillard reaction: furosine, N-ε-(carboxyethyl)lysine (CEL) and N-ε-(carboxymethyl)lysine (CML), together with the dehydroalanine reaction pathway markers; lanthionine (LAN) and lysinoalanine (LAL), as well as lysine itself. The validated method was then used for the absolute quantification of heat-induced protein modifications in model systems of micellar casein and whey protein isolates (MCI and WPI, respectively) in the presence or absence of lactose. As expected, the Maillard reaction markers furosine, CEL and CML increased during the applied heat treatment in the presence of lactose, whereas the dehydroalanine markers, LAN and LAL increased with heating in both MCI and WPI, both in the presence and absence of lactose, although at lower levels in the presence of lactose, confirming the competing state of the two pathways.

## 1. Introduction

Heat treatment processes such as spray-drying, pasteurization and sterilization are commonly used in the manufacture of dairy products. However, heat treatment can induce a range of physical and chemical modifications in the dairy matrix and its components [1]. This can especially affect the molecular properties of proteins, leading to both instant and storage-dependent changes in functional and nutritional values and may limit shelf life due to the introduction of unwanted quality changes. This has been extensively investigated in ultra-high-temperature (UHT) milk, where these modifications can lead to off-flavor [2,3], changes in color [4], lowered nutritional value and digestibility [5]; aggregate formation and sedimentation can also occur [6,7].

The Maillard reaction, proposed to be one of the main contributors behind product changes, is a complex cascade of reactions that occur when proteins are subjected to heat treatment in the presence of reducing sugars. The Maillard reaction can be divided into three stages: early, advanced, and final stages. In the early stage, the presence of reducing sugar in the food matrix or product is required to initiate a condensation reaction between the carbonyl group of the open form of a sugar, such as glucose, galactose, or lactose [8] on one side, and an amino group (e.g., from lysine residues or free amino terminals) on the other side. These can be present as free amino acids or as residues bound into proteins or peptides and will lead to the formation of an unstable Schiff base [9]. This Schiff base then normally further rearranges into the more stable Amadori product. In the advanced stage, this product can be degraded to a reactive α-dicarbonyl compound. This can then react with the α-amino group of free amino acids to form Strecker aldehydes or the nucleophilic side chains of amino acids (most importantly, lysine) bound in peptides or proteins to form different advanced glycation end products (AGEs). Further reactions result in the formation of large groups of cross-linked products, known as melanoidins [10]. To study the progress of the Maillard reaction, different markers can be obtained via acid hydrolysis. Furosine is one such marker of the early-stage Maillard reaction, formed from Amadori products during acid hydrolysis [11]. N-ε-(carboxyethyl)lysine (CEL) and N-ε-(carboxymethyl)lysine (CML) are, alternately, peptide- or protein-bound lysine modifications which are used as markers for the advanced-stage Maillard reaction [12] and derive from the reaction of the α-dicarbonyls glyoxal and methylglyoxal with a single lysine residue. Furthermore, CML can be formed through the oxidation of Amadori products. When glyoxal and methylglyoxal react with two peptide- or protein-bound lysine residues, they form the inter- or intra-protein cross-links, known as glyoxal−lysine dimers and methylglyoxal−lysine dimers, respectively.

Protein cross-linking via the dehydroalanine (DHA) pathway is another processing-induced protein modification that can occur in food protein systems, but is, in contrast to the Maillard reaction, independent of the presence of sugar. It is induced by heat treatment and/or alkaline conditions. Initially, a serine (including phosphoserine and glycoserine), cysteine or a cystine forms into DHA through a β-elimination reaction [13]. The alkene of DHA can then further react via a Michael addition with either a lysine, a cysteine or a histidine residue in proteins and peptides to form the protein cross-links lysinoalanine (LAL), lanthionine (LAN) and histidinoalanine, respectively. No studies have yet described the formation of LAL and LAN involving free amino acids.

Both the Maillard reaction and DHA-mediated cross-linking reaction phenomena are known to be present in long-shelf-life dairy products due to the severe heat treatment used and the presence of reducing sugars [12,14,15]. Although both reactions pathways contribute to the deterioration of dairy products, they have rarely been studied simultaneously, and no knowledge on their interaction exists. The interaction between the Maillard reaction and LAL development is especially relevant because the amino group on the side chain of lysine is a precursor for both pathways. The present study aimed to develop a liquid-chromatography-based method for the simultaneous quantification of Maillard reaction products and protein cross-links using multiple reaction monitoring (MRM) and use this for the absolute quantification of introduced molecular changes in model systems of WPI and MCI heated at severe heat treatment conditions in the presence or absence of sugars. This would enable quantification of the extent of Maillard reaction products and DHA-mediated protein cross-links to understand their impact on food quality.

## 2. Materials and Methods

### 2.1. Materials

Analytical-grade formic acid (FA) was obtained from Honeywell Fluka (Roskilde, Denmark). LC-grade acetonitrile (ACN), LC-MS-grade water, ammonium formate, DL-LAN and L-Lysine-d4 were obtained from Sigma-Aldrich (St. Louis, MO, USA). DL-cystine-d4 was obtained from Cambridge Isotope Laboratories (Tewksbury, MA, USA). Analytical standards of LAL, CEL, CEL-d4, CML, CML-d4, furosine and furosine-d4 were from Iris Biotech GmbH (Marktredwitz, Germany). No commercial standard of histidinoalanine is available; therefore, it was not included.

### 2.2. MCI, WPI and Lactose for Model Studies

For the model study, a casein micellar isolate (MCI) powder, a commercial whey protein isolate powder (WPI, Lacprodan^®^ SP-9224) and a commercial lactose powder (Variolac^®^ 992), all from Arla Foods Ingredients (Nr. Vium, Denmark) (Table 1), were used.

The ingredients were re-dissolved in demineralized water at different concentrations, either individually or in a combination mimicking their concentration in milk (Table 2). To improve the solubility of MCI, it was heated to 50 °C prior to one-way homogenization at 150 bar. Next, all samples were heat-treated in an autoclave (AWA 30) at 121 °C for 0 min, 15 min or 30 min. Samples were placed in the autoclave at 90 °C, and the holding time started when the autoclave reached 121 °C. After heating, the samples were placed on ice for fast cooling. The protein mixtures and their treatments were carried out in duplicates. Six different types and combinations were investigated, representing pure MCI, pure WPI, an MCI + WPI (MCI/WPI) combination, MCI + lactose (MCI + Lac), WPI + lactose (WPI + lac) and MCI + WPI + lactose (MCI/WPI + lac).

### 2.3. Acid Hydrolysis Prior to MRM

Prior to mass spectrometry analysis, acid hydrolysis of the re-dissolved and heat-treated MCI and WPI samples from the model study was carried out. The aim of acid hydrolysis is twofold. Firstly, furosine, as a marker of the early-stage Maillard reaction, is formed from the Amadori product, and secondly, the acid hydrolysis releases the other components of the marker cocktail from their protein bound states. Acid hydrolysis was conducted as previously described [15]. The samples were then stored at −80 °C until analysis. 

### 2.4. MRM Transition Optimization

The commercial standards were made up to a concentration of 10 µg/mL in LC-MS-grade water and the MRM conditions were optimized using the Mass Hunter Optimizer to identify the most abundant precursor and products ions, optimal collision energy and fragmentor voltage. The data obtained were used as settings in the MRM acquisition method (Table 3). 

### 2.5. MRM Quantification

The acid-hydrolyzed samples were analyzed on an LC Infinity 1260 system coupled to a 6460 Triple Quad mass spectrometer (Agilent Technologies, Waldbronn, DE), which operated in MRM acquisition mode. Separation of the compounds released by acid hydrolysis was performed on an Intrada amino acid column (3 mm × 150 mm, 3 µm, Imtrak). The mobile phases consisted of solvent A (100 mM ammonium formate) and solvent B (90% acetonitrile with 0.1% formic acid) using a flow rate of 0.6 mL/min. The column temperature was set to 50 °C. The gradient was as follows: 40–100% A over 4 min, and held at 100% A for 5 min. The flow rate was increased to 0.8 mL/min over 1 min and held for 3 min; then, it returned to the initial condition over 1 min and was held for 4 min re-equilibration. The injection volume was 2 µL. Serial standard concentrations for CEL, CML, lysine and furosine were prepared from 0.0156–2 µg/mL. For LAN, it was prepared from 0.0625–8 µg/mL, and for LAL, it was prepared from 0.25–8 µg/mL. The internal standards were spiked into the mixture before the samples was injected into the LC at a concentration of 0.1 µg/mL of CEL-d_4_, CML–d_2,_ and Lysine-d_4_, 0.2 µg/mL of Furosine-d_4_, and 1 µg/mL of Cystine-d_4_. Quantification was calculated based on the ratio of the analyte and internal standard with MassHunter Quantitative Analysis software (Agilent Technologies). The MRM method was validated for linearity, repeatability, limit of detection (LOD) and limit of quantification (LOQ), as previously described [15]. Initially, the concentration of each compound was obtained as µg/mL, and using the molecular weight of each compound, mmol/L for each compound was calculated. To determine mol compound per mol protein in MCI, the calculations were based on mmol/L of the four caseins in ratios equal to that in milk [16]. To determine mol protein in WPI, the composition of WPI was simplified to consist of only β-lactoglobulin and α-lactalbumin in the ratio between these two as found in milk. 

### 2.6. Statistical Analysis

Differences in the concentrations of processing-induced protein markers in the model study were determined using an analysis of variance with Tukey’s HSD post hoc test using the statistical program R.

## 3. Results and Discussion

### 3.1. Absolute Quantification of Processing-Induced Protein Modifications Using MRM

**Figure 1:**
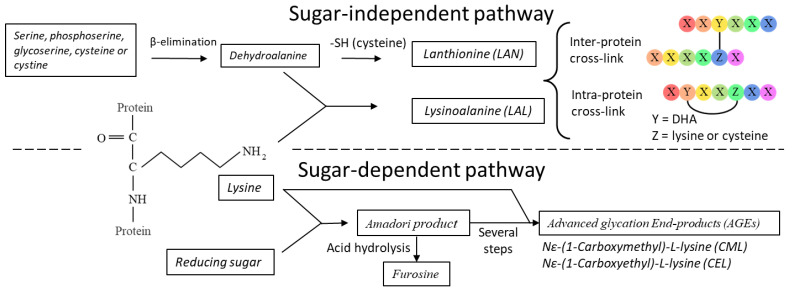
Formation of processing-induced protein modifications from the sugar-independent DHA or sugar-dependent Maillard reaction pathways.

In this study, processing-induced protein modifications were quantified absolutely using LC-MS in MRM acquisition mode. The determined processing markers covered both the sugar-independent DHA-mediated protein cross-linking (LAL and LAN) as well as the sugar-dependent early-stage Maillard reaction marker, furosine, and the two AGEs CEL and CML (Figure 1). In addition, the amino acid lysine was quantified, covering both free lysine and protein-bound lysine. Lysine is a substrate for the initial Maillard reaction and is part of the DHA-induced LAL formation. The developed method was applied to a model study to investigate the impact of severe heat treatment (121 °C for either 0 min, 15 min or 30 min) on processing-induced markers in the casein or whey fraction of milk (using MCI and WPI), either individually or combined in concentrations and ratios comparable to milk (Table 2). Initiation of the Maillard reaction is dependent on a reducing sugar reacting with a nucleophilic amino group; therefore, the present study also investigated how the protein modifications changed according to the presence or absence of lactose.

**Figure 2:**
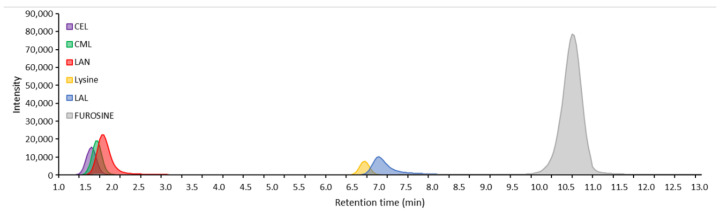
Total ion chromatograms of standard solutions of Nε-(1-Carboxyethyl)-L-lysine (CEL; 62.5 ng/mL), Nε-(1-Carboxymethyl)-L-lysine (CML; 62.5 ng/mL), lanthionine (LAN; 250 ng/mL), lysinoalanine (LAL; 750 ng/mL), lysine (62.5 ng/mL) and furosine (62.5 ng/mL) using LC-MS triple quadrupole in MRM acquisition mode.

Multiple reaction monitoring is the current state-of-the-art method for the quantification of compounds using mass spectrometry. Commercial standards of furosine, CEL, CML, LAN, LAL and lysine of very high purity were used to optimize the MS parameters and the MRM transitions, and were separated using LC (Figure 2). The target ion for quantification was based on the most abundant fragment ion, and the second most abundant ion was selected as the qualifier ion to ensure high specificity of the identified compounds (Table 3). The limit of quantification was identified to be 3.9 ng/mL for CEL, CML, lysine and furosine, 15.6 ng mL^−1^ for LAN and 250 ng mL^−1^ for LAL, which generally showed much lower ionization than the other compounds. At this concentration, the signal-to-noise ratio was >30 for all compounds. Below this concentration, validation parameters were no longer within an acceptable range (±20%). Validation of inter and intra-day accuracy and precision of the calibration curve was determined and was within ±15%, with few exceptions (Supplementary Materials Table S1). For verification of the extraction method, two known concentrations of each compound were spiked into a raw milk sample before or after acid hydrolysis, and their measured concentrations were then compared. Recovery of the compounds was determined based on this measured concentration, and values were within ±10% for all compounds except for LAN at 1 µg mL^−1^ where recovery was determined as 84.5% (Table 4).

### 3.2. Impact of Milk Matrix Components on the Level of Processing-Induced Protein Modifications

MCI, WPI or in combination were subjected to severe heat treatment with or without additional lactose to assess the development in the levels of processing-induced markers depending on protein matrix. Extensive heating was applied to allow the enlargement of differences and to further promote the development of LAN, which has previously only been measured at low levels in milk [12,15]. One adverse effect of the severe heating conditions was that it resulted in WPI forming gel-like structures, which prohibited sampling there. Consequently, no data were obtained for WPI heat-treated at 121 °C for 15 min.

#### 3.2.1. Development in Sugar-Independent Processing Markers in Relation to Milk Protein Matrix

**Figure 3:**
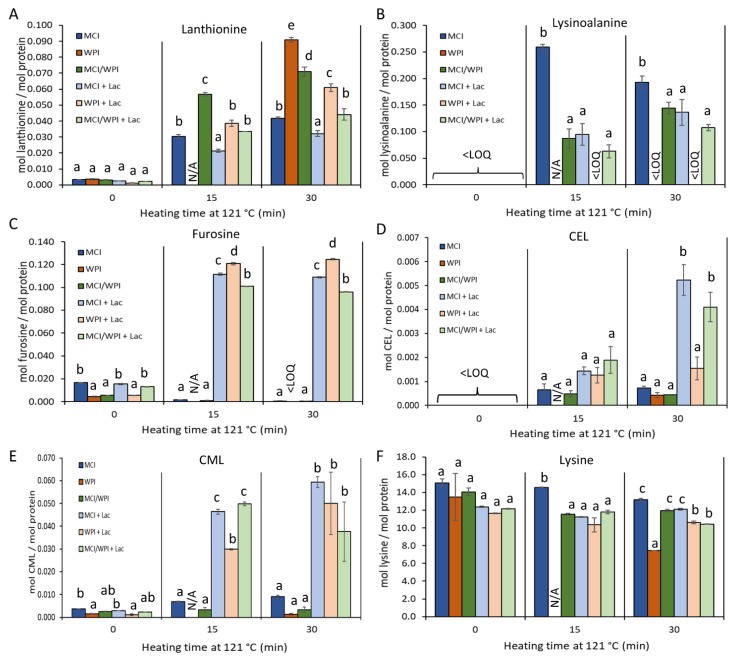
Absolute quantities of DHA-mediated protein cross-links (lanthionine (**A**) and lysinoalanine (**B**)), the Maillard-reaction-derived products (furosine (**C**), CEL (**D**), CML (**E**)) and the amino acid lysine (**F**) in micellar casein isolate (MCI), whey protein isolate (WPI) or in a combination mimicking their ratio in milk (80:20), heated for 0, 15 or 30 min at 121 °C, in the presence or absence of lactose. Different letters indicate significantly different values (*p* < 0.05) in relation to heating (0, 15 or 30 min).

Lanthionine, the cross-linking product of DHA and cysteine, was formed during heating in both MCI and in WPI (Figure 3A). In MCI, the absolute concentration of LAN increased tenfold, from 0.003 to 0.030 mol LAN per mol protein after 15 min of heating, and further increased to 0.042 mol LAN per mol protein after 30 min. When MCI was subjected to heating in the presence of lactose at levels similar to milk, the LAN concentrations significantly decreased to 0.021 and 0.032 mol LAN per mol protein after 15 and 30 min of heating, respectively. The heating of WPI for 30 min increased the concentration of LAN from an initial 0.004 to 0.091 mol LAN per mol protein, being more than twofold higher than MCI heated to the same extent. This could be expected, because the proportion of cysteine amino acid residues are much higher in whey proteins compared with the caseins (being 27 mg/g protein and 4 mg/g protein, respectively) [17]. The majority of cysteine residues in whey proteins are engaged in disulfide bonds (so-called cystine residues). These may also contribute to the increased LAN levels in WPI; it has been shown that disulfide-linked cysteine residues are even more reactive than free cysteine residues. The amino acid sequence of β-lactoglobulin, the most abundant whey protein in bovine milk, contains five cysteine residues, and a-lactalbumin, the second most abundant of the whey proteins, contains eight cysteine residues. In contrast, the mature protein sequences of β- and α_S1_-casein contain no cysteine residues, whereas α_S2_- and κ-casein each contain two cysteine residues. The heating of WPI in the presence of lactose significantly decreased the measured level of LAN as compared with the level obtained by heating without lactose, an effect that was also observed for MCI with and without lactose added during heating. Heating a combination (80:20 *w*/*w*) of MCI–WPI resulted in 0.057 and 0.071 mol LAN per mol protein after 15 and 30 min, respectively. The concentration of LAN was again significantly lower in MCI/WPI heated in the presence of lactose, as compared with the level obtained by heating without lactose.

The concentration of LAL increased in MCI after heating for 15 or 30 min from initially being below quantification levels to 0.259 and 0.193 mol LAL per mol protein, respectively. Likewise, the heating of MCI in the presence of lactose limited the increase in the concentration of LAL to 0.095 and 0.136 mol LAL per mol protein after 15 and 30 min, respectively. Hence, heating in the presence of lactose at levels similar to milk significantly decreased the formation of LAL compared with heating without added lactose. The heating of WPI with and without lactose produced levels of LAL within the detection range, but below the level of calibrated quantification. This difference in obtained LAL levels between the heat-treated casein and whey proteins was somewhat unexpected, with lysine and DHA being precursors for LAL, and lysine being an abundant amino acid residue in both casein and whey proteins (85 mg/g protein and 109 mg/g protein, respectively) [17]. However, in the severe heat treatment (121 °C for 15 or 30 min) conditions employed here, the results show that the reaction of DHA with cysteine/cystine is preferred over that with lysine in WPI. In accordance, prior studies determined cysteine to be more reactive with DHA than lysine [18,19], which could partly explain the obtained results. Despite this, another previous study identified LAL in purified α-lactalbumin heated in dry state at 60 °C for 12–48 h [7]; however, in that study, LAN was not quantified. Differences in heat treatment conditions and in water activity may explain differences in the obtained results. Heating a combination of MCI and WPI resulted in concentrations of LAL below that of heat-treated MCI, which was further lowered in the presence of lactose, although not at significant levels.

LAL has been detected in a variety of processed milk products such as milk powder [15], infant formulae [20] and UHT milk [15,21]; one previous study also confirmed the findings that LAL formation is highest among milk proteins heated in the absence of lactose as compared with heating with lactose [22]. 

In the present study, LAN was also quantified. This DHA-pathway-derived component has only been determined in dairy products in a few studies [12]. The results obtained here show that the level of LAL was much higher than that of LAN in both heat-treated MCI and in the MCI/WPI mix. However, in WPI, LAN was more abundant than LAL. Previous studies found that in UHT milk, the concentration of LAL was much higher than that of LAN [12,15]. 

In the initial phase of the DHA-mediated cross-linking reaction, the reactivity of different amino acid sidechains to undergo β-elimination and form DHA depends on their acidity, with the stronger acid being a better leaving group [23]. Therefore, phosphoserine is more reactive than serine, and because caseins contain a high phosphorylation degree of serine residues, it likely influences their susceptibility to form DHA-mediated cross-linking. 

#### 3.2.2. Development in Sugar-Dependent Processing Markers in Relation to Milk Protein Matrix

Furosine, a marker of the early-stage Maillard reaction, was also identified in all samples prior to heating (Figure 3C). MCI and WPI both represent milk protein products carrying a processing history; therefore, the Maillard reaction was expected to have occurred, as also identified earlier [24]. However, the concentration of furosine prior to the heating employed here was significantly higher in MCI as compared with WPI, which was likely due to the higher content of lactose in MCI as compared with the level in WPI (Table 1). When MCI was heated for 15 or 30 min in the presence of lactose, the concentration of furosine increased from an initial 0.015 to 0.112 and 0.109 mol furosine per mol protein, respectively. The Maillard reaction is initiated by the reaction of a reducing sugar, such as lactose, glucose or galactose, with a nucleophilic amino group; hence, the presence or absence of a reducing sugar, such as lactose, is known to be the key factor determining the occurrence of the Maillard reaction in milk products. The concentrations of furosine were at comparable levels at 15 and 30 min of heat treatment. This is likely due to furosine being a marker of the initial formation of the Amadori product, and the extensive heating used in this study resulted in this initial reaction further progressing to a later stage in the Maillard reaction. A similar result was obtained with WPI and the combined MCI/WPI, where furosine levels were highest when the heating was carried out in the presence of lactose. The highest concentration of furosine after heating was measured in WPI in the presence of lactose.

The concentration of CEL was below the level of quantification in both MCI and WPI when no additional heating was applied. However, CEL was identified in all samples heated for 15 or 30 min, whether lactose was added or not. The identification of CEL in samples without added lactose is likely due to a small level of residual lactose in the MCI and WPI products driving the Maillard reaction. An alternative explanation could, however, be that the Amadori products, measured as furosine levels, confirmed to be present to some extent in these products, have progressed into later-stage Maillard reaction products due to the additional heating performed here (Figure 3D,E). The heat treatment of MCI in the presence of lactose resulted in a CEL level of 0.001 mol CEL per mol protein after 15 min, increasing to 0.005 mol CEL per mol protein after 30 min of heating. The heat treatment of WPI for 15 and 30 min in the presence of lactose resulted in levels of CEL at 0.001 and 0.002 mol CEL per mol protein, respectively. The level of CEL was significantly higher in MCI and MCI/WPI heated in the presence of lactose for 30 min as compared with heating without lactose.

The development in CML content relative to heating with and without lactose followed a similar pattern to furosine. Prior to the employed heating, the initial concentration of CML was significantly higher in MCI than in WPI. By heating in the absence of lactose, comparable concentrations of CML were measured in MCI and WPI, and further being much lower than CML formed in the products heated with the addition of lactose. The heating of MCI in the presence of lactose resulted in levels of 0.046 and 0.059 mol CML per mol protein after 15 and 30 min, respectively. The heat treatment of WPI in the presence of lactose increased CML from 0.001 to 0.030 and 0.050 mol CML per mol protein after 15 and 30 min of heating, respectively. Heating of the MCI–WPI mixture in the presence of lactose lead to an increase in CML from 0.002 to 0.050 and 0.038 mol CML per mol protein after 15 and 30 min, respectively. The formation of CML was much more pronounced than that of CEL, as also observed in previous studies [7].

The level of lysine in MCI, WPI and its 80:20 *w*/*w* mixture was further measured in relation to the heat treatments with and without the presence of lactose. Acid hydrolysis is part of the sample preparation prior to the processing marker cocktail MRM determinations; thus, the measured lysine may represent that originating from proteins, peptides and free lysine and therefore the method does not distinguish between free and bound lysine. The lysine concentration was significantly lower in MCI + lactose after heating compared with MCI heated without lactose (15 and 30 min). The MCI–WPI mixture also showed significantly lower levels of lysine when heated (30 min) with lactose compared with no added lactose. In general, the lowest level of lysine was measured after the longest heating step in all products, which is in accordance with utilization of lysine for both the Maillard reaction and LAL formation (Figure 3F). 

#### 3.2.3. DHA-Mediated Protein Cross-Linking and Maillard Reaction Can Be Competing Pathways

Levels of both LAL and LAN were highest when samples were heated in the absence of sugar. When heating was performed in the presence of lactose, fewer LAL and LAN compounds were detected. In contrast, heating in the presence of lactose increased the formation of the Maillard-related processing markers being part of the MRM cocktail method developed. Similar to DHA-mediated LAL formation, the initial Maillard reaction formation of Amadori products, as well as AGE formation via glyoxal and methylglyoxal, prefer using the amino group on the side chain of lysine as precursors for their reaction. It therefore seems likely that these two pathways may compete for the same substrates. Free amino acids that may be present in the products could be involved in the Maillard reaction formation or in formation of Strecker aldehydes. However, to the best of our knowledge, no studies have investigated whether the DHA pathway may also engage the reactive amino acids in their free forms, in addition to the known protein or peptide-bound reactive residues. Apart from being engaged in LAN formation, cysteine can also be part of reactions related to the formation of AGEs [10], and this could explain the decrease in LAN development when MCI, WPI or the MCI–WPI mixture were heated in the presence of lactose. It could also be speculated that steric hindrance and/or conformational changes from one modification [25] could impact the formation of other modifications, as has been observed in studies of LAL showing that after neighboring sites have reacted, additional cross-links form less readily [26].

In addition to disulfide bond formation [27], both the generation of LAL and LAN, as well as the Maillard reaction (pentosidine, glyoxal-lysine dimer or methylglyoxal-lysine dimer), can induce protein cross-links upon heat treatment. When these occur between protein chains (inter-chain cross-linking), these reactions can contribute to aggregate formation, which is a well-known phenomenon in protein-rich products in relation to heat treatment and storage. Aggregate formation is an unwanted quality change in long-shelf-life dairy products [6]; however, it could also lead to further changes in the digestibility of these food proteins [28,29]. Lysine residues, being part of both Maillard reaction and LAL formation as indicated above, represent a specific cleavage site for the digestive enzyme trypsin, and these modifications are capable of blocking the action of this enzyme during intestinal digestion [30]. Glycation from α-dicarbonyl compounds was further reported to reduce the in vitro digestibility of β-casein and β-lactoglobulin [31]. In line with this, the formation of LAL has also been suggested to decrease the digestibility of proteins [13], although other studies point towards LAL having no impact on digestion. In general, however, few studies have investigated this, and more research is needed [29]. Dietary AGEs can potentially contribute to tissue AGEs [32], and some studies have reported that these compounds could potentially be hazardous after being absorbed in the intestine [33].

In addition to dairy protein, processing-induced protein modifications, presented here as being part of this developed marker cocktail based on MRM technology, are widely present in various protein-based food and feed products. The method developed and demonstrated here has the potential to be applied in the emerging ranges of plant-based and other new alternative protein sources and their derived products. One previous study also developed and demonstrated a method for the quantification of a broad range of Maillard reaction products and DHA-mediated cross-linking formation, including those targeted in the present study [12] using Orbitrap in parallel reaction monitoring mode and two independent chromatographic analysis, whereas this method only uses one. This study quantified the processing-induced protein modification in meat and plasma, as well as milk. However, information about the extent and quantity of processing-induced protein modifications in new alternative protein-based food products remains limited, in spite of their rapid growth markets and increase in availability for consumers.

## 4. Conclusions

This study reports the development of a precise and sensitive MRM-based cocktail method for the simultaneous quantification of processing-induced protein modifications, including DHA-mediated protein cross-linking and Maillard reaction products. The method further quantifies level of unmodified lysine, which is relevant as this amino acid is not only of nutritional importance, but also an essential initial reactant in both Maillard and DHA pathways. The strength of the developed method is that it quantifies derived markers related to both these two pathways leading to processing-induced modifications with potential impacts on both nutritional and quality aspects of food proteins. The formation of LAL and LAN seems to be governed by the proportions of serine, lysine and cysteine residues, as well as their derived modifications, phosphorylation and disulfides in the protein sequences. The initiation of Maillard reaction is highly influenced by the presence of lactose; however, this also indirectly influences the formation of LAL and LAN, because some lysine and cysteine residues are occupied in the Maillard reaction. The competing state between the DHA-mediated cross-linking and Maillard reactions was hence demonstrated by the present study. 

## Figures and Tables

**Table 1:** Powder specifications of micellar casein isolate (MCI), whey protein isolate (WPI) and lactose (*w*/*w*).

	Fat (%)	Protein (%)	Lactose (%)
MCI	1.4	86.5	0.6
WPI	0.1	89.3	0.1
Lactose	0.0	0.2	99.0

**Table 2:** Model study specifications of MCI, WPI and its mixtures with lactose.

	Protein% *(w/v)*	Lactose% (*w/v*)	MCI–WPI Ratio (*w*/*v*)
MCI	3.3	<0.1	100:0
WPI	3.4	<0.1	0:100
MCI/WPI	3.4	<0.1	80:20
MCI + Lac	3.2	4.7	100:0
WPI + Lac	3.5	4.7	0:100
MCI/WPI + Lac	3.2	4.7	80:20

**Table 3:** Parameters used in the acquisition method to quantify Maillard reaction products and DHA-mediated cross-links.

Compound	ISTD ^a^	Molecular Weight (Da)	Ion ^b^	Q1 (*m*/*z*) ^c^	Q3 (*m*/*z*) ^c^	Retention Time (Min)	FV ^d^	CE ^e^
Amino acids								
Lysine		146.2	Target	147.2	84	6.8	63	17
			Qualifier		130	6.8	63	9
Lysine-d4 ^f^	Yes	150.0	Target	151.0	88.0	6.8	67	14
			Qualifier		134.0	6.8	67	6
Cystine-d4 ^f^	Yes	244.3	Target	245.3	153.9	1.9	77	10
			Qualifier		74.0	1.9	77	38
Maillard reaction pathway reporters								
Nε-(1-Carboxyethyl)-L-lysine		218.1	Target	219.1	84	1.6	81	17
			Qualifier		130	1.6	81	9
Nε-(1-Carboxymethyl)-L-lysine		204.1	Target	205.1	84	1.7	81	17
			Qualifier		130	1.7	81	9
Furosine		254.1	Target	255.1	84.1	10.5	91	25
			Qualifier		130	10.5	91	9
Nε-(1-Carboxyethyl)-L-lysine-d4 ^f^	Yes	224.2	Target	223.2	88.1	1.6	100	22
			Qualifier		134.0	1.6	100	10
Nε-(1-Carboxymethyl)-L-lysine–d2 ^f^	Yes	206.1	Target	207.1	84	1.7	73	17
			Qualifier		130	1.7	73	9
Furosine-d4 ^f^	Yes	258.4	Target	259.4	134	10.5	81	9
			Qualifier		89	10.5	81	21
DHA pathway reporters								
Lanthionine		208.2	Target	209.2	119.9	1.9	74	6
			Qualifier		74.0	1.9	74	30
Lysinoalanine		233.3	Target	234.1	84.0	7.0	94	22
			Qualifier		129.9	7.0	94	10

^a^ Internal standard; ^b^ Quantification was based on target ions, whereas qualifier ions were used to validate the targeted compound; ^c^ Q1 are precursor ion masses filtered by the first quadrupole; Q3 are fragment ion masses filtered by the second quadrupole; ^d^ Fragmentor voltage; ^e^ Collision energy; ^f^ Deuterated form of lysine or processing-induced protein modifications.

**Table 4:** Validation parameters (R^2^ values, LOD, LOQ, precision and recovery) for each individual compound included in the LC-MS method.

Compound	Linearity (R^2^)	LOD (ng/mL)	LOQ (ng/mL)	Recovery	
				Low ^a^	High ^b^
Amino acid					
Lysine 147.1 → 84.1	0.9908	1.0	3.9	94.0%	103.4%
Maillard reaction products					
Nε-(1-Carboxyethyl)-L-lysine 219.1 → 84.1	0.9921	1.9	3.9	104.4%	93.3%
Nε-(1-Carboxymethyl)-L-lysine 205.1 → 84.1	0.9980	1.9	3.9	105.1%	104.9%
Furosine 255.1 → 84.1	0.9926	1.0	3.9	104.0%	91.3%
DHA-mediated cross-links					
Lanthionine 209.2 → 119.9	0.9926	7.8	15.6	104.1%	84.5%
Lysinoalanine 234.1 → 84.0	0.9971	125.0	250.0	105.2%	112.3%

^a^ CEL, CML, lysine, furosine = 0.03 µg/m, LAN = 0.125 µg mL^−1^ and LAL = 0.5 µg mL^−1^; ^b^ CEL, CML, lysine, furosine = 0.25 µg/mL, LAN = 1 µg mL^−1^ and LAL = 4 µg/mL.

## Supplementary Materials

The following supporting information can be downloaded at: https://www.mdpi.com/article/10.3390/foods11070897/s1, Table S1: Intra- and inter-day precision and accuracy of each individual compound included in the LC-MS method.
