# Supplementary material for: Influence of Lactose on the Maillard Reaction and Dehydroalanine-Mediated Protein Cross-Linking in Casein and Whey
Source: Foods. 2022 Mar 22;11(7):897. doi: 10.3390/foods11070897 (PMC8997915; doi:10.3390/foods11070897)
Supplement: Supplementary file 1 [file foods-11-00897-s001.zip › foods-1560751-supplementary.pdf]

Supplementary table 1. Intra- and inter-day precision and accuracy of Nε-(1-Carboxyethyl)-L-lysine (CEL), Nε-(1-Carboxymethyl)-L-lysine (CML), Lanthionine, lysine, lysinoalanine and furosine

Concentration (ng/mL)	Intra-day precision (%CV, n = 3)	Intra-day accuracy (%, n = 3)	Inter-day precision (%CV, n = 6)	Inter-day accuracy (%, n = 6)
**CEL**				
3.9	126.5	11.6	111.1	16.8
7.8	95.0	3.0	96.0	7.6
15.6	89.0	2.3	94.6	4.0
31.3	90.6	4.2	93.2	4.7
62.5	91.8	2.2	95.5	4.4
125	98.5	1.9	101.4	24.4
250	98.9	2.8	101.4	4.2
500	98.9	2.1	98.6	5.4
1000	103.3	3.1	103.2	4.8
2000	99.3	2.0	99.0	5.1
**CML**				
3.9	123.9	3.8	123.1	17.4
7.8	96.9	7.3	103.9	14.0
15.6	92.4	6.2	95.3	4.1
31.3	94.9	4.3	98.3	4.1
62.5	91.7	3.9	94.3	2.7
125	102.1	2.7	94.9	1.8
250	100.2	0.7	100.8	2.2
500	97.9	3.8	96.0	3.3
1000	103.0	1.2	101.5	2.2
2000	99.4	1.6	98.2	2.5
**Lanthionine**				
15.6	122.0	0.4	107.9	15.3
31.3	93.8	9.3	91.7	9.7
62.5	96.2	6.9	91.0	5.2
125	94.3	3.8	91.0	6.7
250	88.4	7.5	89.9	6.3
500	94.4	8.5	98.4	16.0
1000	96.9	2.4	101.6	5.6
2000	96.0	2.2	99.4	7.8
4000	105.1	3.0	103.5	6.9
8000	99.3	3.3	94.3	5.3
**Lysine**				
3.9	111.7	14.4	119.1	20.6
7.8	75.6	2.8	105.0	13.6
15.6	88.7	2.0	98.6	9.9
31.3	98.3	2.7	97.1	8.4
62.5	94.6	5.3	94.9	2.1
125	97.6	1.5	98.7	2.9
250	98.8	2.7	97.1	3.3

500	97.3	3.6	97.3	8.4
1000	104.0	2.9	102.6	3.0
2000	99.3	3.6	99.6	4.4
**Lysinoalanine**				
250	83.8	6.6	96.7	5.0
500	88.4	18.5	94.6	7.7
750	93.0	8.5	93.0	6.0
1000	87.8	6.4	91.6	7.2
2000	97.2	2.6	97.0	1.9
4000	113.3	6.4	104.0	10.2
6000	110.7	6.4	98.2	6.2
8000	91.2	2.5	94.8	7.2
**Furosine**				
3.9	115.5	11.0	105.4	4.2
7.8	95.5	10.5	95.9	4.6
15.6	93.9	1.5	93.5	2.2
31.3	94.4	2.9	94.9	3.6
62.5	93.8	1.2	96.1	4.4
125	96.3	2.9	100.6	3.4
250	101.7	3.0	102.1	4.5
500	97.0	1.0	98.7	4.2
1000	103.2	1.6	103.0	3.7
2000	99.5	3.0	99.8	3.9
